# Exploring the Perspectives of Professionals on Providing Intimate Partner Violence Services to Women With Disabilities

**DOI:** 10.1177/10778012221137916

**Published:** 2022-11-20

**Authors:** Fredinah Namatovu, Jens Ineland, Veronica Lövgren

**Affiliations:** 1 Epidemiology and Global Health (EpiGH), Centre for Demographic and Ageing Research (CEDAR), Umeå University, Umeå, Sweden; 2 Department of Social Work, Umeå University, Umeå, Sweden

**Keywords:** accessibility, disability, intimate partner violence, services, professionals

## Abstract

This study explored the experiences and perceptions of professional service providers offering services to women with disabilities exposed to intimate partner violence (IPV). Eighteen in-depth interviews were conducted with service providers working in health care, social work, the police, women’s shelters, and the Centre for Violence Against Women. Our findings suggest that providing adequate IPV services to women with disabilities requires coordination and collaboration. IPV services were organized around five overarching themes: finding services; assessing the risk; identification; protection and care; and becoming independent. This approach was helpful for women who faced disability-related challenges in accessing IPV services.

## Background

Intimate partner violence (IPV) is the most common form of gender-based violence reported to occur in all countries, all cultures, and at every level of society (Garcia-Moreno et al., 2006). Evidence suggests that IPV disproportionately affects girls and women with disabilities worldwide (Brownridge, 2006; Hughes et al., 2012; Jones et al., 2012). Increased vulnerability to IPV among women with disabilities varies by type and degree of disability (Hughes et al., 2012). The intersectional forces such as limited material resources (Robinson, Frawley, et al., 2021), the constraining social environment (Mueller et al., 2019), stigma, discrimination, lack of social support, and dependence on others for long-term support (Brownridge, 2006; Nosek et al., 2001; Shah et al., 2016) play a significant role in increasing the vulnerability of women with disabilities.

IPV is a major public health threat that increases physical and mental health problems, including injuries, depression, post-traumatic stress disorder, and poor sexual and reproductive health (Ellsberg et al., 2008; Garcia-Moreno et al., 2006; Thomas et al., 2008). Unfortunately, the devastating consequences of IPV tend to continue long after the end of an abusive relationship. Women separating in the context of victimization have a high risk of physical and mental health problems and report several economic, psychological, and social barriers to help-seeking (Logan & Walker, 2009). Adequate IPV support plays a critical role in minimizing the negative health and social impacts related to exposure to IPV (Evans & Feder, 2016; Rose et al., 2000). Timely and efficient IPV responses deter future IPV incidents, prevent fatal outcomes, and foster recovery from the physical and emotional damage caused by IPV (Broidy et al., 2015; WHO, 2013). A provider–client relationship that facilitates trust, choice, and agency is key for successful violence services (Robinson, Valentine, et al., 2021).

Despite the well-documented benefits of IPV services, women with disabilities report difficulties in accessing these services due to infrastructural barriers such as inaccessible facilities (Feder et al., 2006; Robinson, Frawley, et al., 2021). Additionally, women with communication and intellectual disabilities report difficulties in disclosing abuse and in seeking out help (Fraser-Barbour Ellen et al., 2018). Women with disabilities that need assistance in their day-to-day life report that searching for and finding IPV services is problematic, especially in situations where the caretaker is both the partner and the perpetrator (Evans & Feder, 2016). Other reported barriers to accessing IPV services include a lack of confidence in the providers, the provider's failure to screen for abuse, and services that are not adapted to the specific needs of people with disabilities (Evans & Feder, 2016).

### IPV Services in Sweden

According to the WHO, IPV is defined as any behavior within an intimate relationship that causes physical, psychological, and sexual harm to those in the relationship (Garcia-Moreno et al., 2006). Sweden does not have an equivalent concept for IPV, rather national policies frame this in terms of “violence within close relationships,” implying that it does not necessarily have to involve an intimate partner (SOU, 2014). Sweden recognizes violence within close relationships as a legal and social problem, and explicitly declares it a public health matter whose mitigation requires coordinated actions between multiple societal institutions (Gunnarsson et al., 2007; SOU, 2014). This paper uses the term “IPV services” as an umbrella term, referring to various services, support, and care offered to individuals exposed to IPV. We specifically focus on services offered by professionals working within health care, social services, the police, women’s shelters, and the Centre Against Violence (centrum mot våld). We describe below how these services function in Sweden.

Within the healthcare sector, the National Board of Health and Welfare (NBHW) emphasizes the prevention of IPV using early detection of those at risk through routine healthcare inquiry about violence and preventative work with perpetrators. The health sector operates at the regional level within the 21 regions in Sweden (SOU, 2014). In the region of Västerbotten, where the current study was conducted, healthcare work on IPV is organized within the Care Program (Vårdprogram). The Care Program provides an overview of IPV knowledge and general routines for executing IPV services.

IPV services provided by social workers are available for all citizens and are regulated by the Swedish Social Services Act (SoL, 2001, p. 453). The Swedish Act concerning Support and Services for Persons with Certain Functional Impairments (LSS, 1993, p. 387) gives people with certain disabilities more extensive rights. IPV work within social services is implemented in all 290 municipalities in the country, steered by a local municipality Social Welfare Committee (National Board of Health and Welfare, 2014). Social services can be organized differently by the different specialized units or as integrated services (Perlinski et al., 2010). The Directive (SoL, 2001, p. 453) states that organizations should indicate who is responsible for investigating, making decisions, and following up cases concerning victims of violence following the Social Services Act.

The Swedish police operate within the Swedish criminal justice system and recognize IPV as a prevalent global problem (Strand et al., 2018). Members of the criminal justice system, specifically police and prosecutors, are tasked with the responsibility of responding to acts of IPV and protecting the exposed from further violence (Belfrage et al., 2012). Each police district has its specific unit working exclusively with IPV. The police use structured violence risk assessment tools to prevent future IPV. These tools guide decision making on case prioritization and execution of appropriate risk management strategies such as safety talks, alarm packages, contact with social services, and shelters (Rikspolisstyrelsen, 2010).

To a large extent, women's shelters operate as voluntary organizations although occasionally they receive government grants and/or municipal reimbursement for services, and some may have employees. Most women's shelters belong to the National Organization for Women's and Young Women's Shelters in Sweden (ROKS) or the Swedish Association of Women's Shelters and Young Women's Empowerment Centres. Each of these organizations includes about 100 centers across the country (Antilla et al., 2006; Helmersson & Jönson, 2015).

In Västerbotten county, the Center Against Violence operates as an organizational driving force for work on violence against women and children. The Center Against Violence works through structured government cooperation, consultative activities, and method development networking with the Västerbotten County Council, Umeå Municipality, the Police Authority, the Public Prosecutor's Office, the Forensic Medicine Agency and Umeå University, and with the Children's Home and the Women's Peace Clinic. Vårdprogram för arbete med våld i nära relationer (Care program for work with violence in intimate relationships) (regionvasterbotten.se).

Research concerning the perspectives of service providers directly working on violence against women is gradually increasing (Robinson, Frawley, et al., 2021; Wathen & MacMillan, 2003; Young et al., 2019). However, the literature reveals that services oriented toward combatting IPV are fragmented, lacking mutual integration (Autiero et al., 2020). In addition, most studies focus on women in general and not specifically on women with disabilities (Mueller et al., 2019; Namatovu et al., 2018). It is vital to consider IPV service provision among women with disabilities given that disability intersects with other identities, such as gender, socio-economic status, and ethnicity, known to affect access and utilization of IPV services (Mueller et al., 2019; Robinson, Valentine, et al., 2021). This study builds on existing findings and adds to the limited body of research in this field by exploring the views and experiences of IPV service providers on providing IPV services to women with disabilities in Sweden.

## Aim

The objective of this study was to explore how service providers experience and perceive the organization of IPV services for women with disabilities.

## Methods

### Study Design

This study applied a constructivist grounded theory approach that is based on the principles of symbolic interactionism to explain a phenomenon (Charmaz, 2014). Symbolic interactionism addresses how people create, interpret, endorse, or change meanings based on actions and interactions experienced in their daily lives (Charmaz, 2020). Individuals make sense of the world through actively interacting with other people and with themselves based on the symbolic meaning given by society (McDonald & Schreiber, 2001). The interaction between the researcher and the participant is considered naturally dynamic, involving regular engagement in an interpretative process. For this study, constructivist grounded theory enabled the generation of the substantive theory to explain the previously undescribed subject of how existing services address the needs of women with disabilities exposed to IPV. The perspectives of different service providers were considered pertinent as they were best positioned to explain their roles, actions, and interactions with women with disabilities that sought their services. Our team aimed to capture the experiences of IPV service providers offering services to women with disabilities. Multiple dissemination methods were used to reach a diverse group of service providers in terms of geography and nature of services. We circulated information about the study via contacts with various service providers, community-based organizations, disability-related websites, and the social media of individuals active in the disability community.

All participants consented to participate both verbally and by providing a signature. Before the start of each interview, the interviewer reminded participants of their right to withdraw participation, refuse to answer questions they did not want to answer, and that they could ask that the recording be turned off during any part of the interview. The interviews were conducted in a single session primarily by the second author except for a few interviews conducted by the third author. The first author joined and listened to several of these interviews. The original plan was to interview participants face-to-face, but due to the COVID-19 outbreak, we opted for digital interviews. All interviews were conducted over Skype or Zoom; the meetings lasted (on average) 60 min and the audio from them was recorded and then later transcribed.

### The Participants

This study included 18 service providers working in health care, social work, the police, women’s shelters, and the Center Against Violence. Participants from health care included a physician, physiotherapists, counselors, and psychologists. The social workers were employed within the local government and offered counseling to clients who experienced violence and abuse. The service providers working at shelters were trained to offer counseling and support at safe houses, which are secret locations where women can flee from their abusive partners. The service providers at the Center Against Violence described their role as offering support to women who experienced IPV and their children. The police officers worked in specific units that target violence, and their primary role was to investigate whether a crime has been committed. However, the police also worked in partnership with other organizations and service providers at the municipality level, victim support groups, and with voluntary organizations that provide sheltered housing (Rikspolisstyrelsen, 2010). Providers were at varying stages of their career, ranging from having worked for 2 to more than 20 years. Even though we aimed at interviewing both male and female IPV service providers, only one man participated. Also, nearly all the providers had never provided IPV to men with disabilities, thus we framed our study as the perspective of professionals on providing services to women with disabilities.

### Interview Content

An open-ended interview guide was used, and it covered a series of topics related to the process used by women with disabilities to find IPV services, the structure of services, routines in place, and the nature of collaboration in providing services to women with disabilities. Additional follow-up questions were asked based on the participants’ accounts.

### Data Analysis

The transcripts were coded using MAXQDA software version 20.2, and the analysis followed Charmaz's constructivist grounded theory. The initial step in the analysis involved carefully reading the interviews to capture the overall substance of the content. Data analysis began by open coding, examining each line of data, and defining actions or events within. Each new transcript was coded and compared to previously analyzed interviews, allowing for the refinement of existing codes and the development of new codes. This was followed by collapsing the initial codes into smaller lists of focused codes based on similarities, for example, see Table 1.

**Table 1.** Example of the Analysis Process Showing the Movement From Raw Data to Theoretical Coding.

Raw data	Initial coding	Focused coding	Theoretical coding
It happens quite often that we get contacts mediated to us via a counselor at a school for example or the hospital, but it can also be through the social services or the police. Then some people have said that they want to contact us in that case, that they want us to call or that professionals ask them if they can give them our phone number.	Finding services through collaborating with professionals like: - School counselors - Hospitals - Social services - Police	IPV services providers	Coordinating and collaborating to provide IPV services
One comes here in different ways … you can write to us on our website or email, but you can also write on social media, we also have a chat nowadays, but you can of course call.	Finding services on her own: - Websites - Email - Social media - Phone calls	Disabled woman exposed to IPV	

*Note.* IPV = intimate partner violence.

**Figure 1.**
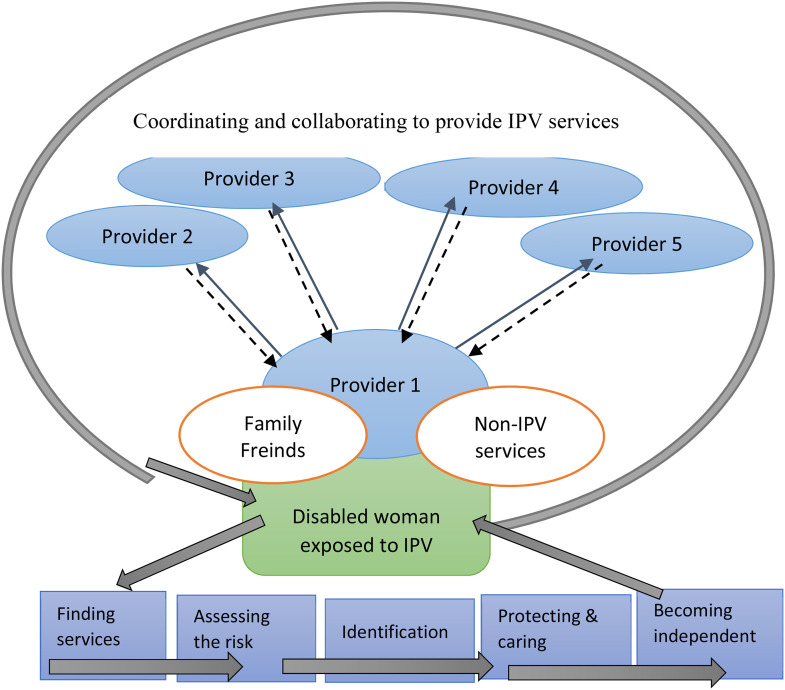
A Theoretical Model Illustrating the Service Providers’ Description of the Collaboration and Coordination Process of Providing IPV Services to Women With Disabilities and the Five Themes Taken by Providers Together With Women With Disabilities. *Note.* IPV = intimate partner violence.

Visual models were created to better organize and conceptualize the focused codes. Focused codes were compared and related to each other for theoretical coding to develop core concepts for the theoretical model. One core category identified describes how IPV services were organized for women with disabilities. Several iterations of the model were produced before the final model was developed. Finally, an integrated grounded theoretical model was achieved, describing the core process of providing IPV services to women with disabilities (Figure 1).

## Results

Providing services to women with disabilities subjected to IPV was found to be a dynamic process that could be understood by using the theoretical framework of “coordinating and collaborating to provide IPV services.” The process of IPV service provision was characterized by five interrelated themes: finding services; assessing the risk; identification; protection and care; and becoming independent (Figure 1). Letters are used next to the quotes presented in place of names to protect participants’ identities.

*Coordinating and collaborating to provide services:* Identifying a person or an agency to coordinate the process of service provision was an important element in IPV service provision to women with disabilities. The coordinator helped in building bridges between the disabled woman and the service provision system. Well as all providers perceived coordination as important and more effective if the providers took on this role, it was only those working at women's shelters that often took on the responsibility of coordination. “We usually become some kind of spider-in-the-net, which is usually very much appreciated, that she gives us permission to speak with all her other contacts” (LL). “I became some kind of intermediary between the social services and this woman” (CR). In some cases, the coordination role could be assumed by someone from the civil society, such as the contact person if a disabled woman was living in a service home, “The people I have met who, for example, live in service homes, they often have a contact person in the service home these take the role of being the spider-in-the-net” (AK).

Several forms of collaboration were described, the first form was between the disabled woman and the initial service provider she established contact with. This collaboration helped to understand the IPV problem, how and where to get help, “We learn things, we find together with the woman things that would work in everyday life that make it easier to move forward in what we are going to talk about, about the exposure to violence” (KN). It was important that a woman felt trusted by the first provider she encountered. Trust aided the continuation of services and in establishing contact with other service providers. Providers also described the multi-sectoral collaboration as crucial in providing effective services to women with disabilities. “One hopes to create other contact paths” (RA). Whom to collaborate with was assessed on a case-by-case basis. For example, if a woman was referred by social services, it was the social services that assessed her needs and engaged other parties to work with her. “Yes, if there was already ongoing violence, occurring right now, we talk about the Center Against Violence, where they have the Center against violence on children, women, and men” (AC). Collaboration with other providers played a very important role in organizing and keeping track of the services offered by the different providers. “It feels important to talk to each other in the network…. I think that collaboration becomes so important, otherwise, it's so easy for someone else to think someone else has done something. Sometimes, we have both the neurology clinic, geriatrics, and health centers and I think that you then clarify who does what” (MK). “But we work together. We do not do it in parallel tracks … to avoid everyone doing the same thing and someone is always dissatisfied and there is confusion” (SK).

Aside from other IPV service providers, in some cases, collaboration requires working with non-IPV service providers. Including non-IPV service providers helped to ensure that a disabled woman received comprehensive support even beyond IPV services. Determining which non-IPV providers to include was based on the assessment of the individual needs of the woman. Non-IPV providers included caretakers, housing companies, migration offices, and childcare services. “We help to try to bring in different help and call different authorities and caregivers and, with preschool classes and housing and all those practical things…. We keep in touch with the Swedish Social Insurance Agency and the Swedish Migration Board” (LL).

Sometimes providing adequate IPV services to women with disabilities required collaboration with the woman's social support network. “You talk to the patient and … a sister or brother or any relative … so that they know what it's like” (MK). Social networks were important, particularly when the women with disabilities lived in isolation or had limited social interaction. Making others in their circle aware was important in ensuring continuity in seeking services. However, providers noted that this was only feasible if a disabled woman already had supportive family members and/or friends.

Another form of collaboration at a structural level is described as cross-sectoral collaboration for capacity strengthening. This involved working with the different institutions to organize joint staff training sessions on violence, develop training materials, assessment of organizing IPV work. “We have produced a joint handbook, or administrative support, for collaboration. All parties have their administrative support. But we have developed one that will apply to us in collaboration. That handbook consists of a bit of what is violence?” (KH).

### Themes in Collaboration for Providing Services to Women With Disabilities

Service providers described the collaboration system of service provision for women with disabilities as characterized by five interrelated themes, including finding services; assessing the risk; identification; protection and care; and becoming independent.

#### Finding IPV Services

Participants indicated that one of the key components of providing IPV services to women with disabilities was being able to find IPV services. Women accessed services via a professional agency, third-party agency, or own agency.

Finding services via a professional agency was by far the largest pathway described as used by women with disabilities to access IPV services. Examples of professionals include social workers, doctors, nurses, psychologists, police, and personnel at women's shelters. Providers came across disabled women in need of IPV services during their day-to-day work. “We get in touch with people with disabilities through our work” (ALG). Most women came in contact with the providers seeking other services, then the provider inquired about IPV. Gradually, women shared their experiences and eventually got access to IPV services. This process involved collaboration between the disabled woman and the first provider. If a woman needed other services, the provider would initiate a referral or a collaboration with other providers. However, the inquiry about IPV was related to the provider's primary responsibilities. Providers who primarily offered IPV counseling inquired regularly about IPV among women with disabilities. These providers also informed clients about other available IPV services elsewhere. Providers whose primary work did not relate directly to IPV rarely inquired about IPV, unless there was a visible sign of a woman being in a current abuse situation; otherwise, providers opted for referrals “to send them to other types of staff who are much better trained and equipped for such questions than we are” (IM).

The third-party agency was the second most common path used by women with disabilities to gain access to IPV services. Here, finding IPV services required a joint effort between the disabled woman, the third party, and the provider. Often, the “third party” contacted the IPV service provider after witnessing abuse or suspecting that a disabled woman was experiencing IPV; “A staff member at care home calls and says that this person has told me that she has been the victim of a rape” (IM). The third party either contacts the service provider directly or assists the disabled woman to contact the providers. Examples of those belonging to the third-party category include caregivers, heads of home care centers, mentors, or a “Goodman”—described as a publicly commissioned guardian appointed to oversee the financial affairs and judicial matters affecting citizens with intellectual disabilities (Mallander, 2015). “It can also be a goodman or a close relative, but more often a goodman or mentors if they are these younger people, or personal assistants, or housing supervisors who flag the situation” (AGN).

*Own agency:* Service providers revealed that on rare occasions, the disabled woman initiated the search for IPV services. In these cases, the woman would file a complaint to the police, contact a social service center, or visit a women's shelter or a health care facility. “Of course, some have no problem getting here and being here themselves” (AK). However, providers noted that it was rare for women with disabilities to take the initiative, as illustrated by this provider, “The absolute most unusual way is that they seek out for services” (AN). Providers described that when women with disabilities sought out IPV services, this was often followed by a series of abusive patterns and violence from their intimate partner. The women with disabilities who sought out support on their own had to be aware that abuse was not acceptable, aware of the existing IPV services, and able to communicate and move physically.

#### Assessing the Risk

Once contact was established between the disabled woman and the provider, the providers assessed whether the woman was at risk of experiencing IPV by inquiring about violent experiences in her intimate relationship. Some providers used standardized IPV screening tools, although several acknowledged difficulties, as most tools were not adopted for clients with disabilities. In such cases, providers would collaborate with others that had special skills to facilitate communication, “to have someone with you to kind of make sure with sign language…. I have to ask for help from others” (AC).

The process of assessing the risk of violence was described differently by various providers. For women who directly sought IPV services, the process was mainly focused on assessing the magnitude of risk and how to minimize it. For women who did not come directly to seek IPV services, the adopted approach was dependent upon the primary task of the providers. Providers whose primary tasks included providing IPV services described routinely assessing IPV risk. “The first thing I do when I meet a client is to inquire, where I then ask about their experience with intimate partner violence, if they have experienced it and what it looks like” (ALG).

Providers described assessing the risk as a gradual process because clients did not often mention exposure to IPV on the first visit. “One does not say this in the first conversation that it is about violence. Instead, perhaps one is applying for housing, or … support for substance abuse or something else. Then after meeting the provider for some time, the experience of violence begins to come out” (AGN). Providers described that establishing rapport and building trust were the key facilitators that enabled women to open up about their IPV experiences. “You also have to be very careful not to scare people somehow when you ask questions. You have to create an environment that enables her to tell you herself” (DH).

Providers whose primary task did not include offering IPV services did not routinely inquire about IPV. These took an opportunistic approach, inquiring about IPV in clients perceived to be at high IPV risk. Some working in health care often inquired about IPV when women presented with health problems that were highly indicative of abuse. For example, having bruises, wounds, unexplained fractures, and chronic somatic problems. Providers that primarily worked with children described inquiring about IPV if a mother missed her child's appointment frequently or when a child developed emotional or behavioral problems. Social workers who did not directly work on IPV inquired about abuse if, during a couple's counseling session, the partner appeared controlling. Even providers who did not routinely inquire about IPV generally viewed routine IPV assessment as important. Time constraints and a heavy workload were the reported barriers to routine IPV inquiry, “One doesn’t have time to talk so much. I think it is good, but sometimes it also takes time … You send them to a psychologist or to someone who can take care of them” (DB).

#### Identification

Identification was described in two ways: identifying the IPV experienced by the disabled woman and identifying the type of disability that a woman had. Professionals expressed the importance of identifying IPV as early as possible. They framed identifying IPV early in their interaction as important to ensure timely IPV interventions and provision of services. IPV identification could take several forms, for clients that directly sought out IPV services, this was straightforward. However, most clients did not directly look for IPV services. In such cases, service providers relied on their professional judgment to identify IPV. They paid attention to non-verbal communications and less obvious signs. “People might meet someone at the front desk, maybe someone who leaves blood samples at the lab … Someone notices during this visit that there is something that is not right. Then one can say that I think there is much more going on” (DH). Community social workers who offered financial advice described that they inquired about IPV if a woman was continuously struggling to maintain her economy, “if it appears that they have finances issues” (MK). The process of identifying IPV was considered gradual, requiring first the client to gain trust in the provider.

Another form of identification was regarding disability. Sometimes the providers encountered difficulties to identify that the client had a disability. “But it can also be that someone comes in and makes a report … where you do not know that there is a disability. It may also be that when we meet the person for the first time, you cannot say what kind of disability this person has” (CV). In such cases, providers emphasized the importance of collaborating with the client's social network, such as caretakers, goodman, family, and friends, to clarify the type of disability a client has. “It is so important to talk to a close relative or goodman or staff at the accommodations … for clients who have a very hard time talking, they cannot describe violence or sexual violence” (CV).

#### Protection and Safety

Once IPV was identified, providers worked toward ensuring protection and safety. Protection involved engaging in activities that shielded the disabled woman from further abuse, “We first identified whether the person needed immediate protection or support … an assessment must be made as to whether it can be stepped up, whether the person can be taken out of the home, whether there is danger” (KH). Utilizing close collaboration with a woman's close network, providers helped the woman move to a safe place, “Usually they need someone … a close friend who sort of tears them out of there. They have a hard time making their own decisions” (CR).

Once short-term safety was achieved, the provider and the disabled woman worked jointly to establish long-term safety. This included finding permanent accommodation and increasing knowledge concerning safety precautions, that is, keeping home doors locked, establishing who the visitor was before opening the door, and reducing contact with the abuser. “It became a job to arrange a peephole on the door, strategies for what to do when someone rings the doorbell, who should you open up to? So today she makes them wait, she locks the door always. She does not open now when someone comes. If one stands outside her door, they have to send an SMS and say that ‘I’m standing outside the door, you can open now’” (ALG). Ensuring a woman's safety includes collaborating with several service providers and supportive family members. Providers aimed at personalizing services to address the specific needs of each woman, this required engaging the woman in the process of identifying her needs. The woman's needs were constantly reviewed, and her consent was continuously sought.

When children were involved, the dynamics of protection significantly changed; cases were handled faster to ensure that children were removed from an abusive environment, and investigations remained open for a long time. “When there are children in the picture, … they do not close down their cases. But the group working with children, for example, must have the case open longer” (AK). When children are involved, providers were compelled by the law and professional obligation to report the violence and in such cases, the woman's consent was not a priority.

#### Becoming Independent

Providers described that a large component of service provision included supporting the disabled woman to become autonomous and to take initiatives independently. Municipality social workers primarily working with individual, and family financial matters described their primary task as that of offering financial support, housing, and support. Services that help women gain more independence. “Some women who come here for the first time and no longer live with their husbands, it's about money, it's about housing and it's about support. It's the first three” (AK). Providers worked together with the woman to alleviate her financial concerns, if the woman had no source of income or if she depended on the abuser. In some cases, women had their income but needed support to obtain a bank account, apply for a credit card, write a CV, or begin searching for employment.

Providers also supported women with disabilities to acquire self-defense skills and learn to be in charge of their bodies. “We have also supported a group for young girls with intellectual disabilities to promote their self-defense, tell them that one has a right to say no, you have the right to know what it means to have the right to your own body, as part of making things clear” (LL). Becoming independent also involved working together with the woman to develop individual strategies to assess whether she was at risk of IPV, identify ways of reducing IPV risk, and how to seek help independently. Becoming independent also included engaging women with disabilities in decision making regarding IPV services, what type of services they needed, and when and where to seek these services.

Even though becoming independent was considered important, in some cases, the type of disability created challenges. For example, women with communication and/or intellectual disabilities faced unique difficulties to exercise independence when seeking IPV services. The following provider describes a struggle between a client's independence and getting help:

she calls me when he has just left, and she is really upset and cries a lot and screams and it was very difficult to understand her because this woman also has a language impairment. So I go to her and we talk and she is very sad and very upset and I talked to her about reporting this to the police … she wanted to go there by herself, she does so…. Then time passes for a while, and she calls again and again when she is with the police, and it is very difficult for her to understand what they are asking her. (AS)

Such challenges created difficulties in establishing autonomy and in accessing IPV services.

## Discussion

The current study provided the perspectives of IPV service providers on the way IPV services are organized for women with disabilities. Coordination and collaboration in providing IPV services were identified as the suitable model for women with disabilities. This approach had five overarching themes; finding services; assessing the risk; identification; protection and care; and becoming independent.

Providers perceived coordination and collaboration as essential for providing comprehensive IPV services to women with disabilities. Having a central person helping the women coordinate the various services was key because many with disabilities often lacked the personal agency to navigate the system on their own. The coordinator could be a service provider or a family/friend. However, in describing the provider's role as coordinator, there was a mismatch between this perception and the current practice. Many providers perceived this role as a time constraint and preferred to refer clients to other providers. The findings in this study show the importance of having coordinating bodies (peoples, units, etc.) that are equipped with the intersectional understandings in IPV services and put into consideration the perspectives and experiences of people with disability (Robinson, Frawley, et al., 2021; Robinson, Valentine, et al., 2021).

In judging how successful service provision was for women with disabilities, some providers relied heavily on the performance of other actors where they referred cases. Reliance on the performance of other providers might have implications for the quality of services. If each provider assumes that the next provider will address the needs of the woman, consequently the needs might remain unmet. This further points to the need for a coordinator to keep track to ensure access to IPV service, to avoid what Frawley, Dyson, and Robinson (2017) identified as a “clash of cultures,” about woman's personal and social values, which may represent barriers to accessing services. Reflecting on these intersectional dimensions in IPV also offers opportunities for improvement and development.

Even though this study focused on the role of professional services providers, we found evidence that supportive informal networks such as family members and friends of a disabled woman were important in coordinating and collaborating IPV services. This was crucial in facilitating access to multiple services from different actors. Moreover, a Swedish report indicated that abused women with disabilities were more likely to turn to family and friends for support compared to formal support (Handu, 2007). Similarly, another study found that abused women were more likely to confide in someone close to them than confiding in professional service providers, 69% were more likely to talk to a family or friend, compared to 34% who confided in a doctor, or nurse and 30% who confided in a mental health counselor (Goodman et al., 2003). The role of informal support networks needs further exploration.

Assessing IPV risk was an important step for identifying abused women with disabilities and those at high IPV risk. This finding supports previous evidence showing that assessing IPV leads to women's receipt of interventions and improved health (O'Doherty et al., 2015). This also further emphasizes the need for the health sector, police, and social work to promote the importance of inquiring about IPV and screening for exposure to violence (Feltner et al., 2018; Messing & Thaller, 2014; Sprague et al., 2016). However, providers lacked a consistent approach for assessing IPV risk in women with disabilities. In addition, providers lacked tools and training adapted to address the different forms of disabilities. Research is required to investigate how to adapt existing tools and training to the needs of women with disabilities and how to organize capacity strengthening for providers of IPV services to women with disabilities. Proper IPV tools are shown to enhance the facilitation of communication between providers in other settings such as the police, prosecution, and judicial responses to IPV, as they provide a consistent language regarding risk factors and the measurement of risk (Messing & Thaller, 2014).

In the current study, providers that did not consider IPV service provision as their primary task pointed to time constraint as a major barrier for routinely inquiring about IPV among women with disabilities. This is a missed opportunity, for example, in health care, women with disabilities are considered high-level consumers due to the high rates of mental disorder, exposure to multiple forms of abuse, and high rates of child sexual abuse (Rees et al., 2011; Trevillion et al., 2012). Women with disabilities report limited social interaction; contact with a service provider may be the only opportunity for the woman to disclose abuse and to get access to IPV interventions (Feder et al., 2009).

Literature shows that successful implementation of IPV interventions is dependent on the extent to which the organizational settings promote the implementation of the required changes (Klein & Sorra, 1996). Increasing environmental support for IPV screening among women with disabilities might help improve their access to IPV services, especially targeting providers who do not primarily provide IPV services (Campbell et al., 2001). In accordance with the evidence by Robinson, Frawley, et al. (2021), our study suggests that intersectional awareness and being able to address broader issues of accessibility in IPV services, in addition to disability-specific needs, hold the potential to match available services to the needs of a woman with disabilities.

This study further showed that increasing the independence of clients was a major milestone in reducing IPV among women with disabilities. Interventions that address the abused women's housing insecurity and financial autonomy were viewed as key in enabling a disabled woman to leave an abusive partner and gain autonomy. Women's shelters were viewed as an effective emergency housing intervention that provided a starting point for establishing safety and enabled women to live independently. This finding is in line with studies indicating that shelter services and innovative interventions constitute effective housing strategies for abused women (Goodman et al., 2003).

## Conclusion

This study identified a theoretical framework of coordinating and collaborating to provide IPV services as instrumental in facilitating women to navigate the disability-related barriers. The process of providing IPV services to women with disabilities constituted five overarching themes: finding services; assessing the risk; identification; protection and care; and becoming independent. Reflecting on these themes helps to ensure routine inquiry about IPV, establish rapport, and build trust. Factors that encourage women with disabilities to open up about their IPV experiences. The significance of early identification of IPV was also emphasized, as this would help ensure timely intervention and provision of IPV service in form of protection and care; and resources that increase the women's independence. Improved access to IPV services among women with disabilities will help mitigate the negative health and social consequences associated with IPV.
